# Case Report: Twin Pregnancy Gives Birth to a Girl with Partial Trisomy 21 Mosaicism after *in vitro* Fertilization and Embryo Transfer

**DOI:** 10.3389/fgene.2021.740415

**Published:** 2022-02-03

**Authors:** Zhenglong Guo, Bing Kang, Dong Wu, Hai Xiao, Leilei Hao, Bingtao Hao, Shixiu Liao

**Affiliations:** ^1^ Henan Provincial Key Laboratory of Genetic Diseases and Functional Genomics, National Health Commission Key Laboratory of Birth Defects Prevention, Medical Genetic Institute of Henan Province, Henan Provincial People’s Hospital, People’s Hospital of Zhengzhou University, Zhengzhou, China; ^2^ School of Medicine, People’s Hospital of Henan University, Henan University, Zhengzhou, China; ^3^ Department of Biology, University of Pennsylvania, Philadelphia, PA, United States; ^4^ School of Basic Medical Sciences, Cancer Research Institute, Southern Medical University, Guangzhou, China

**Keywords:** IVF-ET, NIPT, chromosome defects, twin pregnancy, down syndrome, case report

## Abstract

**Objective:** To report a rare case in which an IVF-ET twin pregnancy gave birth to a partial trisomy 21 chimera girl.

**Design:** Case report.

**Setting:** University hospital.

**Patient:** A girl with partial trisomy 21 mosaicism after *in vitro* fertilization and embryo transfer.

**Interventions:** *In vitro* fertilization (IVF) and embryo transfer (ET).

**Main Outcome Measure:** Karyotype analysis, Copy Number Variation sequencing (CNV-seq), stLFR-WGS, and Short Tandem Repeat (STR) analysis.

**Results:** Being assisted with IVF and EF technology, the couple successfully gave birth to twin sisters at 37 weeks of gestational age. The NonInvasive Prenatal Testing (NIPT) and Nuchal Translucency (NT) examination showed no detectable genetic abnormalities during pregnancy. However, the younger infant displayed growth retardation and feeding difficulties after birth, which was not observed in her twin sister. Further genetic counseling and diagnosis suggested that she is a Chimera with complex partial trisomy 21. The stLFR-WGS assay showed multiple CNV variations in Chr21 and STR analysis confirmed the paternal origin of the additional fragments.

**Conclusion:** It is rare for IVF-ET-assisted twin pregnancy to give birth to a girl with a complex combination of abnormal Chr21, which might result from paternal chromosome rearrangement during meiosis and mitosis.

## Introduction

After the first “test-tube baby,” Louise Brown was born after conception by *in vitro* fertilization experiment (IVF) in 1978 (Steptoe and Edwards, 1978). IVF- and intracytoplasmic sperm injection (ICSI)-based Assisted Reproductive Technology (ART) development has rapidly soared (Bonduelle et al., 2005; Dyer et al., 2016; Fishel, 2018; Saito et al., 2018). Since 1978, millions of babies were born by ART, marking the technology a widespread alternative for treating human infertility in the past decades (Meldrum, 2013; Sunderam et al., 2015; Johnson, 2019). However, despite the prevalence of ART, it is unclear if IVF or ICSI increased the risk of congenital disabilities in newborns. Aneuploidy is the most common genetic abnormality and considered the leading cause of implantation failure, miscarriage, and congenital disabilities (Nagaoka et al., 2012; MacLennan et al., 2015). The high frequency of aneuploidy in IVF-produced embryos was thought to be one of the main reasons affecting the implantation and pregnancy rates (Franasiak et al., 2014).

To address the issue, researchers developed the preimplantation genetic testing for aneuploidies (PGT-A), which can analyze the embryonic chromosomal status, do embryo selection prior to transfer, and thereby allow implantation of genetically normal embryos (Brezina and Kutteh, 2015). Many methods have been successfully applied in the PGT-A process, such as fluorescence *in situ* hybridization (FISH) on fixed cells, array comparative genomic hybridization (aCGH), digital polymerase chain reaction (dPCR), single-nucleotide polymorphism (SNP) array, real-time quantitative PCR (qPCR), and next generation sequencing (NGS) (Sato et al., 2019). Nevertheless, PGT-A was mainly proposed for advanced maternal age (AMA), defined as ≥ 37 years; repeated implantation failure (RIF); history of recurrent miscarriage (RM); and severe male factor infertility (Rubio et al., 2019).

Trisomy 21, or Down syndrome, is one of the most commonly occurring aneuploidies caused by the presence of all or part of an extra chromosome 21. Trisomy 21 is manifested by multiple phenotypes, including intellectual disability, congenital heart defects, and muscle hypotonia (Hecht and Hook, 1996; Antonarakis et al., 2004; Mazurek and Wyka, 2015; Antonarakis, 2017; de Graaf et al., 2017; Reeves et al., 2019; Bull, 2020; de Graaf et al., 2021). Advanced maternal age, environmental factors, as well as meiotic and mitotic errors are the main risk factors for trisomy 21 (Antonarakis et al., 1992; Torfs and Christianson, 2003; Allen et al., 2009; Ghosh et al., 2009; Nagaoka et al., 2012; Hunter et al., 2013; Coppede, 2016; Gruhn et al., 2019; Keen et al., 2020). Here, we present a rare case about a young couple with normal karyotypes who underwent IVF and ET assisted pregnancy. The twin pregnancy gave birth to a partial trisomy 21 female with a complex karyotype and multiple CNVs. Further analysis suggested a paternal origin of the extra chromosome 21 fragment.

## Materials and Methods

### *In Vitro* Fertilization and Embryo Transfer

*In vitro* fertilization (IVF) was carried out based on the standard long protocol. First, pituitary suppression was achieved by administering 3.75 mg triptorelin acetate (Ipsen Pharma Biotech, Paris, France). When the patient reached the criteria for pituitary suppression (Fang et al., 2020; Hu et al., 2020), ovarian stimulation was initiated with gonadotropin (Gonal-F, Merck Serono, Geneva, Switzerland; Puregon, Organon, Oss, The Netherlands). When one primary follicle diameter was ≥20 mm and at least two follicles reached 18 mm, hCG (Ovitrelle, Merck Serono) was injected to trigger oocyte maturation. Follicle aspiration was conducted through transvaginal ultrasound 36–38 h after hCG administration. Retrieved eggs were fertilized and checked after incubation with the sperm from the husband, in which two good-quality cleavage embryos were picked up and transferred transcervical under ultrasound guidance. Biochemical pregnancy was determined by the serum β-Hcg concentration increase at 14 days after ET, and clinical pregnancy was defined by the presence of a gestational sac by abdominal ultrasound at 35 days after ET.

### Copy Number Variation Sequencing

Fifty nanograms of DNA extracted from peripheral blood was fragmented. DNA libraries were constructed by end filling, adapter ligation, and PCR amplification. DNA libraries were then subjected to massively parallel sequencing on the NextSeq 500 platform (Illumina, San Diego, CA) to generate approximately 5 million raw sequencing reads with genomic DNA sequences of 36 bp in length. The hg19 genomic sequence was used as a reference. A total of 2.8–3.2 million reads were mapped using the BurrowseWheeler algorithm. Mapped reads were allocated progressively to 20-kb bin sizes from the p to q arms of the 24 chromosomes. Counts in each bin were compared between all test samples run in the same flow cell to evaluate copy number changes using previously described algorithms. Sprinkle, a comprehensive tool developed by BerryGenomics, was used for CNV calling. The CNVs were interrogated against publicly available databases, including Decipher, Database of Genomic Variants (DGV), 1,000 genomes, and Online Mendelian Inheritance in Man (OMIM), and their pathogenicity was assessed according to the guidelines outlined by the American College of Medical Genetics (ACMG) for interpretation of sequence variants.

### Karyotype Analysis

Peripheral blood samples from the proband were cultured using the standard technique. G banding assay was used for analysis, and more than 100 metaphase chromosome images were captured and investigated for the Chimera.

### stLFR Whole Genome Sequencing

Peripheral blood genomic DNA extracted from the proband was quantified using the dsDNA BR assay on a Qubit fluorometer (Thermo Fisher Scientific, Waltham, MA, USA). The stLFR technology used Tn5 transposase to construct DNA libraries according to the standard protocol using the MGIEasy stLFR Library Preparation kit v1.1 (PN: 1000005622)*. In brief, the transposon integrated DNAs were hybridized with magnetic beads containing multi-copy molecular barcodes by the principle of DNA double-strand complementation adapter and sequenced on the BGISEQ-500 sequencer. The original data was filtered and compared to the human reference genome (GRCh37/Hg19) to obtain the initial alignment result. Picard tool was applied to remove duplicate reads, and GATK (v4.0.3)’s HaplotypeCaller was used for base quality recalibration. Based on the comparison results, the evaluation indexes such as the sequencing depth, coverage, and comparison ratio of each sample were counted. Copy number variation (CNV) was detected using the self-developed LFR-CNV software.

### Short Tandem Repeat Analysis

To identify the origin of the extra Chr21 fragment, peripheral blood-derived DNA samples of the father, mother, and proband were prepared. Four Chr21 specific STR markers (D21S2052 in 21q21.3, D21S1246 in 21q22.2, D21S11 in 21q21.1, and Penta D in 21q22.3) were analyzed using PowerPlex 21 HS genotyping system (Promega, Madison, WI, USA) according to the manufacturer’s instructions. The Sequence Information for Selected STR Systems could be found on the STRBase (https://strbase.nist.gov/seq_info.htm). Amplification mixture with 5 μl of PowerPlex 21 5× Master Mix, 5 μl of PowerPlex 21 5× Primer Pair Mix, and 5 ng of DNA were conducted in GeneAmp PCR System 9,700 Thermal Cycler (Applied Biosystems, Carlsbad, CA, USA). PCR conditions were as follows: 96°C for 1 min; 94°C for 10 s; 59°C for 1 min; 72°C for 30 s, 30 cycles in total; and 60°C for 10 min. PCR products were detected by capillary electrophoresis and analyzed using the GeneMapper ID ver. 3.2 software (Applied Biosystems).

### Case Report

In 2019, a 26-year-old woman diagnosed with infertility caused by tubal obstruction was assisted with reproductive technology (ART) and *in vitro* fertilization (IVF) (two embryos were transferred). Her husband was 28 years old, and the routine examination of semen quality showed normal results. The karyotype analysis showed that the couple had normal karyotypes (46, XX, and 46, XY) without any detectable deletion, duplication, translocation, or inversion. Genetic counseling results showed no family history of genetic diseases. Combined, the couple was suggested to do the IVF-ET without PGT-A. *In vitro* fertilization (IVF) was carried out based on the standard long protocol mentioned in the method. After embryo transfer, serum β-Hcg and abdominal ultrasound demonstrated successful clinical pregnancy. The mother received NIPT (Berry Genomics, Beijing, China) and Nuchal Translucency (NT) examination at 12 + 4 weeks gestation, which was generally applied to screen chromosome aneuploidies, especially for Trisomy 21 (T21). In this case, ultrasound results showed two viable fetuses with normal NT value, in which the F1 fetus was 1.4 mm and F2 fetus was 2.4 mm. Meanwhile, NIPT indexes were below 0.5, indicating a low risk for T21, T18, and T13. At 22 + 2 weeks of gestation, color Doppler ultrasound was used to detect the development of the two fetuses, and no abnormality was observed.

**FIGURE 1:**
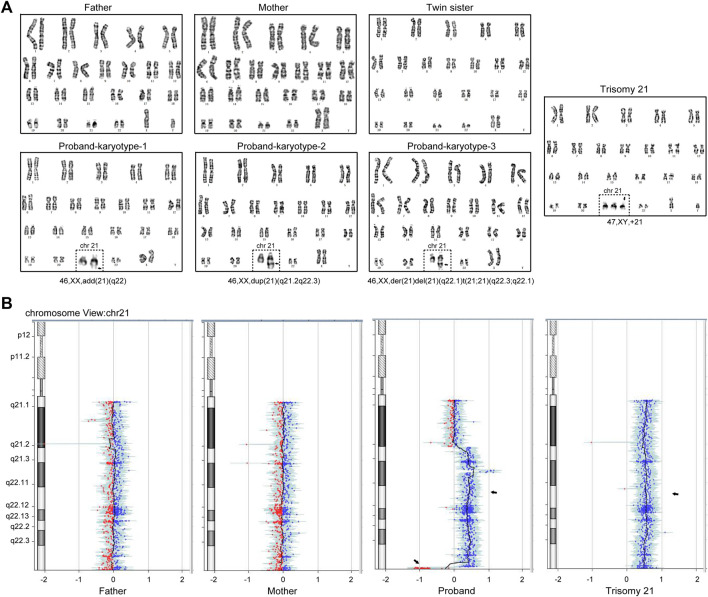
A couple gave birth to a Chimeric child with a partial trisomy 21 after IVF and EF. **(A)** Giemsa staining and analysis of the cultured peripheral blood mononuclear cells in the family members, in which the proband exhibits the combination of three karyotypes: 46,XX, add (21) (q22), 46,XX, dup (21) (q21.2q22.3) and 46,XX, der (21)del (21) (q22.1)t (21; 21) (q22.3; q22.1). **(B)** Copy number variation sequencing (CNV-seq) showed the copy number of Chr21 (q21.2-q22.3) was 2–3. The Trisomy 21 patient karyotype and CNV-seq results were used as positive control.

The twin pregnancy gave birth to two girls at 37 + 1 week of gestation by cesarean section in 2020, with no hypoxia or birth trauma. However, one neonate weighed 2.9 kg, and was observed to exhibit hypotonia and feeding difficulties 10 days after birth. Further ultrasonic examination demonstrated a 4.0-mm ventricular septal defect, patent foramen ovale, and tricuspid regurgitation in the heart. On the contrary, the sister did not show any abnormalities. Full karyotyping was performed on the twin sisters after genetic counseling. The result showed that the proband is a Chimera with a partial trisomy 21 karyotype, while the sister has a normal 46, XX karyotype. G-banding from peripheral blood cells of the patient showed a complicate karyotype of 46,XX,add (21) (q22)[25]/46,XX,der (21)del (21) (q22.1)t (21; 21) (q22.3; q22.1)[36]/46,XX,dup (21) (q22.1q22.3)[32] (Figure 1A). The CNV-seq result consistently showed the copy number of Chr21 from q22.1 to q22.3 was 2–3 (Figure 1B).

**FIGURE 2:**
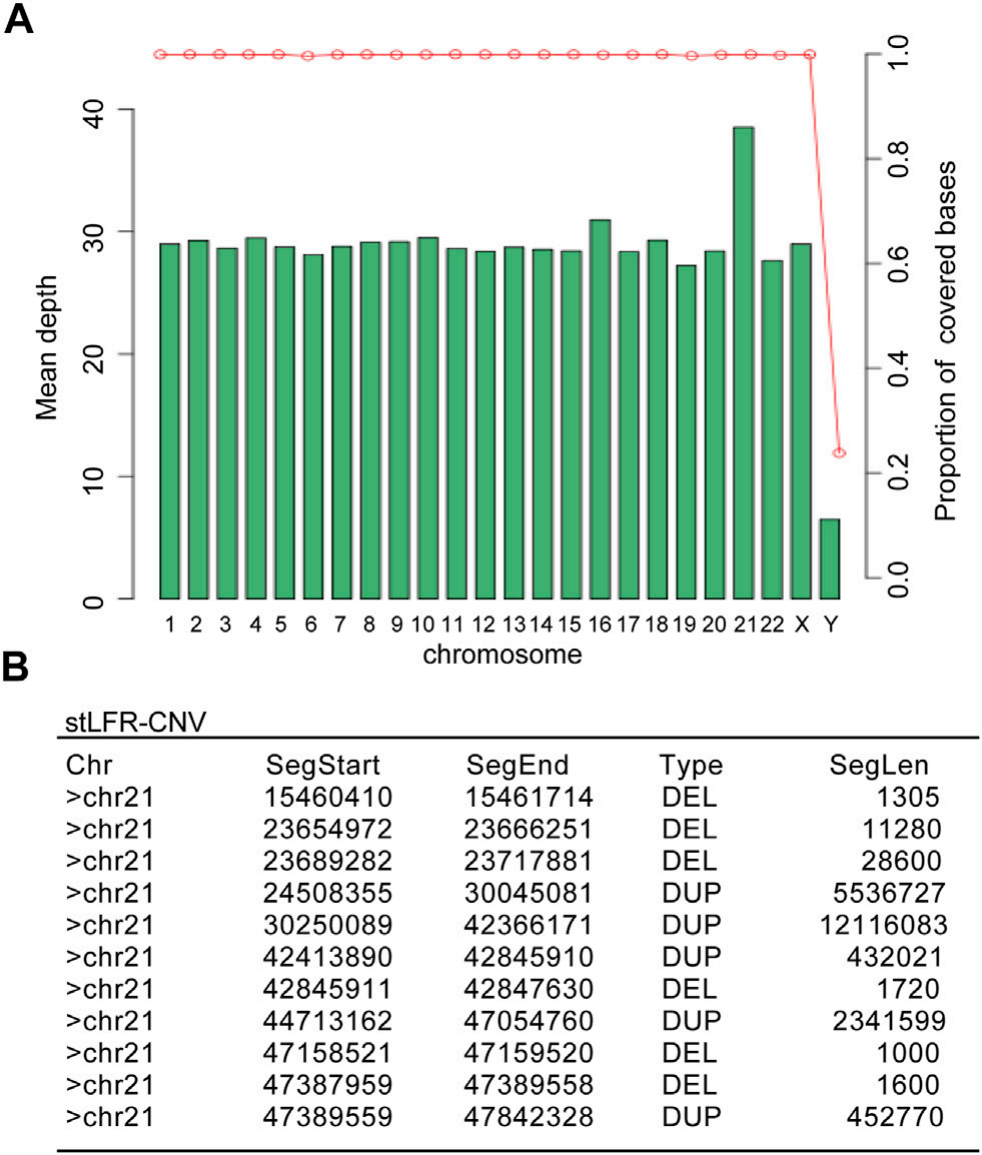
The stLFR whole-genome sequencing result showed multiple duplications and deletions in the proband’s Chr21. **(A)** The mean depth distribution in different chromosomes of the proband. **(B)** Detailed copy number variations (CNVs) in the proband’s Chr21 summarized from the genome sequencing comparison.

**FIGURE 3:**
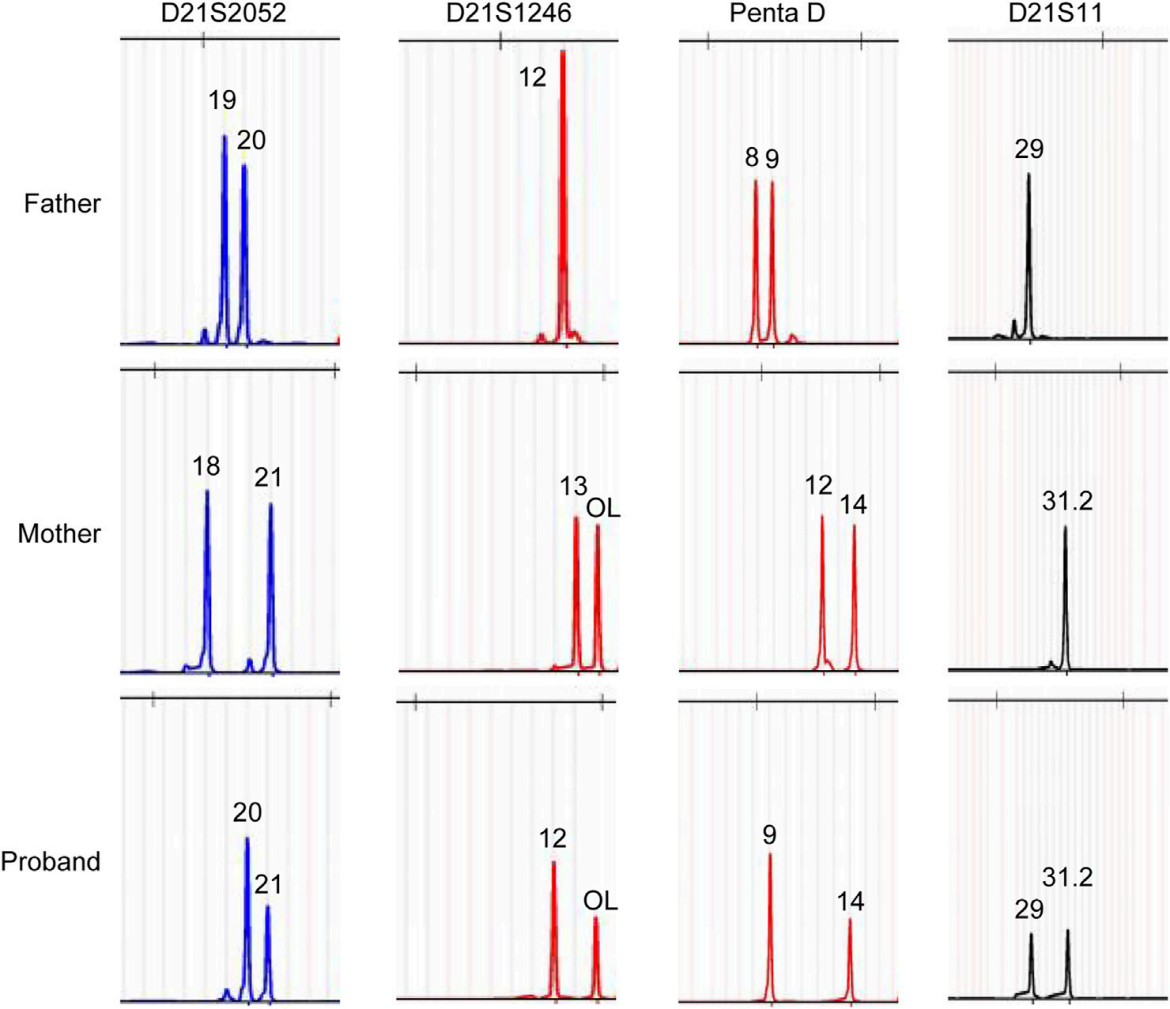
Polymorphic Chr21 specific DNA marker analysis showed a paternal origin of the extra chromosome 21 fragment.

To better understand the Chr21 re-arrangement in this case, we applied the stLFR-WGS developed by BGI to detect the precise breakpoints and CNVs of Chr21. Figure 2 listed the candidate CNVs of Chr21, including multiple larger duplication and small deletion from q22.1 to q22.3. To trace the origin of the additional Chr21 fragment, a Short Tandem Repeat (STR) analysis with four Chr21-specific DNA markers was performed using the DNA extracted from the girl and parental blood samples. The result showed a paternal origin of the extra chromosome 21, indicating the paternal origin of the partial trisomy 21 (Figure 3).

To track the development of the affected twin and establish the relationship between the Chr21 mosaicism and disease phenotype, we suggest a long-term follow-up of the patient, especially for the Chr21 aneuploidy-related manifestations, such as intellectual development and nervous and cardiovascular system function, which would be helpful for the clinical management of similar cases.

## Discussion

Emerging evidence has suggested the increased risk of congenital disabilities, including chromosome abnormalities and development disorders, in fetuses conceived with ART methods (Chen and Heilbronn, 2017; Sammel et al., 2018; Yu et al., 2018; Luke et al., 2020; Wen et al., 2020; Luke et al., 2021; Wang et al., 2021). The results from our case report indicate that IVF-ET pregnancies need more attention and careful prenatal screening. Fetal nuchal translucency (NT) thickness, maternal serum biochemical markers (such as PAPP-A and free β-hCG), as well as NIPT utilizing high-throughput methods for detecting free placental DNA (cfDNA) are the most commonly applied prenatal screening methods, which could provide a risk assessment for common autosomal aneuploidies (Giroux et al., 2021; Kimelman and Pavone, 2021; Merriel et al., 2021; Qi et al., 2021; Suzumori et al., 2021). Although there is a high prediction rate of NIPT in singletons, twin pregnancies are dizygotic (DZ), which reduces the serum markers and cfDNA concentrations, increasing the risk of a missed diagnosis (Struble et al., 2014).

In this case, the young couple was assisted with *in vitro* fertilization (IVF) and embryo transfer (IVF) because of tubal obstruction induced infertility. Unexpectedly, successful twin pregnancy gave birth to a partial trisomy 21 mosaicism and a normal child. It is worth mentioning that nuchal translucency (NT) examination and NIPT failed to screen this mosaicism fetus, which was finally confirmed through karyotyping assay and CNV-seq analysis. The result showed a complex combination of duplication from q22.1 to q22.3 and distal absence and fused chromosome in Chr21. It suggests that the present prenatal diagnosis regulations may not meet the detection of the Chr21 chimera in this case.

The stLFR-WGS was applied to analyze the potential breakpoints in the Chr21 (Wang et al., 2019). Multiple candidate breakpoints and formed CNVs from q22.1 to q22.3 were discovered, suggesting that IVF-introduced breakpoints may be enlarged during the meiotic and mitotic process *in vitro*, thus attributing to the complex Chr21 mosaicism in the proband. Short tandem repeat (STR) analysis with Chr21-specific DNA markers demonstrated that the extra Chr21 segments originated from the father. The proband exhibited hypotonia and feeding difficulties, and further ultrasonic examination identified Cardiac dysplasia, which the extra Chr21 may cause. We will continuously track the development of the child, including her nervous, immune, cardiovascular, and other bodily systems.

Considering some reported cases of twin pregnancy gave birth to children with trisomy 21 following IVF, the abnormal Chr21 may result from the IVF-ET process (Chaliha et al., 1999; Dunn et al., 2001; Zeng et al., 2003; Lavery et al., 2008). It seems like traditional methods of nuchal translucency (NT) and NIPT have limitations in detecting the trisomy 21 mosaicism in a twin pregnancy. Meanwhile, some articles have revealed a high incidence of aneuploidy and mosaicism in embryos from young couples undergoing IVF (Baart et al., 2006). Therefore, the preimplantation genetic testing for aneuploidies (PGT-A) in young couples taking IVF-ET should be considered, although more data is needed to make a definitive statement.

## Data Availability

The data that support the findings of this study is available from the corresponding author upon reasonable request.
